# Supplementary material for: Microbiota Profile of the Nasal Cavity According to Lifestyles in Healthy Adults in Santiago, Chile
Source: Microorganisms. 2023 Jun 22;11(7):1635. doi: 10.3390/microorganisms11071635 (PMC10384449; doi:10.3390/microorganisms11071635)
Supplement: Supplementary file 1 [file microorganisms-11-01635-s001.zip › microorganisms-2440628-supplementary.pdf]

microorganisms

MDPI

*Article*

# Microbiota Profile of the Nasal Cavity According to Lifestyles in Healthy Adults in Santiago, Chile

**Daniela Toro-Ascuy [1,†], Juan P. Cárdenas [2,3,†], Francisco Zorondo-Rodríguez [4], Damariz González [2], Evelyn Silva-Moreno [1,‡], Carlos Puebla [1,§], Alexia Nunez-Parra [1,‖], Sebastián Reyes-Cerpa [2,3,*] and Loreto F. Fuenzalida [1,*]**

[1] Facultad de Ciencias de la Salud, Instituto de Ciencias Biomédicas, Universidad Autónoma de Chile, Santiago 8910060, Chile; daniela.toro@uautonoma.cl (D.T.-A.); evelyn.silva@umayor.cl (E.S.-M.); carlos.puebla@uoh.cl (C.P.); alexia.nunez@uchile.cl (A.N.-P.)
[2] Centro de Genómica y Bioinformática, Facultad de Ciencias, Ingeniería y Tecnología, Universidad Mayor, Santiago 8580745, Chile; juan.cardenas@umayor.cl (J.P.C.); damigonzalez38@gmail.com (D.G.)
[3] Escuela de Biotecnología, Facultad de Ciencias, Ingeniería y Tecnología, Universidad Mayor, Santiago 8580745, Chile
[4] Departamento de Gestión Agraria, Facultad Tecnológica, Universidad de Santiago de Chile, Santiago 8910060, Chile; francisco.zorondo@usach.cl
* Correspondence: sebastian.reyes@umayor.cl (S.R.-C.); lfuenzalidai@uautonoma.cl (L.F.F.); Tel.: +56-2-25189260 (S.R.-C.); +56-2-23036662 (L.F.F.)
† These authors contributed equally to this work.
‡ Current Address: Vicerrectoría de Investigación, Universidad Mayor, Avenida Manuel Montt 376, Providencia, Santiago 7500994, Chile.
§ Current Address: Instituto de Ciencias de la Salud, Universidad de O'Higgins, Rancagua 2841935, Chile.
‖ Current Address: Physiology Laboratory, Biology Department, Faculty of Science, Universidad de Chile, Santiago 7800003, Chile.

## Supplementary Materials

*Supplementary Figures*

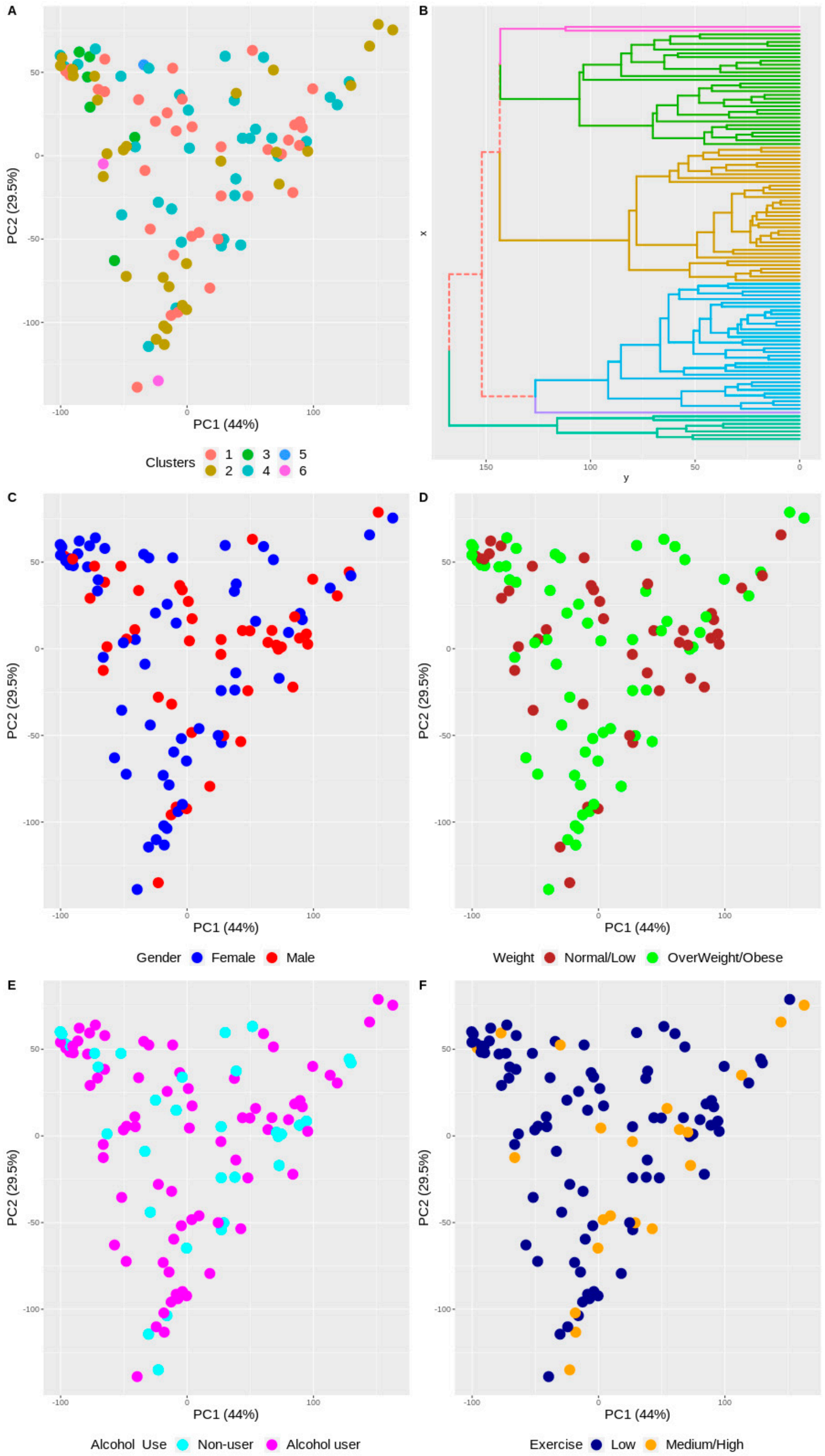


**Figure S1.** Clustering (PCA) profile of the nose microbiome. The PCA profile (A) with the top 6 clusters coloured per each sample. Those clusters were observed in the dendrogram (B) created from the average hierarchical clustering with the "hclust" instruction. Samples in the PCA profiles were

also coloured according to their categories according to sex (C), nutritional status (D), alcohol Use (E) and physical activity (F).

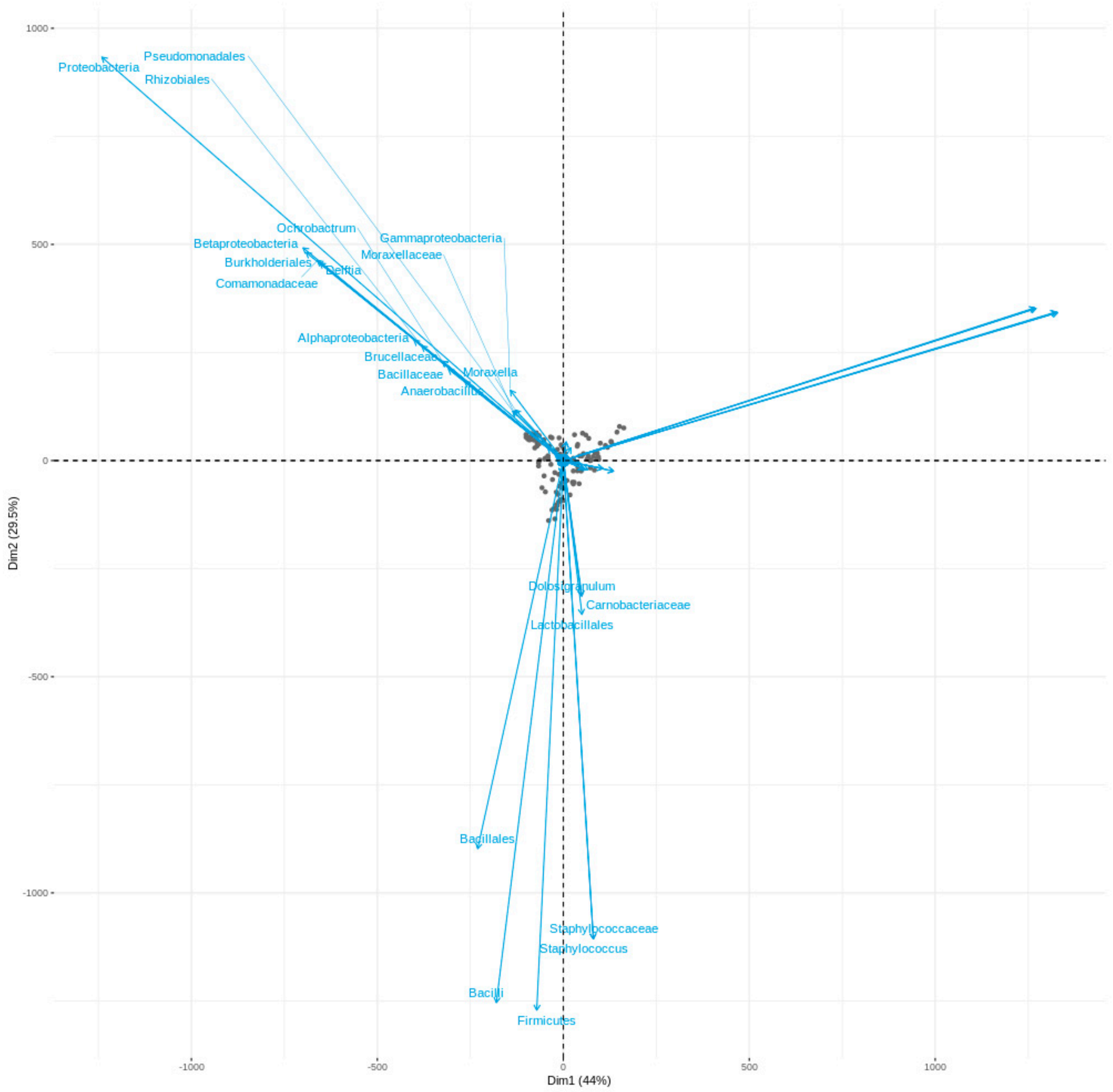


**Figure S2.** Variant representation influencing PCA behavior of the samples. The arrows represent the different variants (in this case, taxonomic groups) defining the behavior of the samples (dots) across the plane. This PCA is the same as the shown in Figure S1.

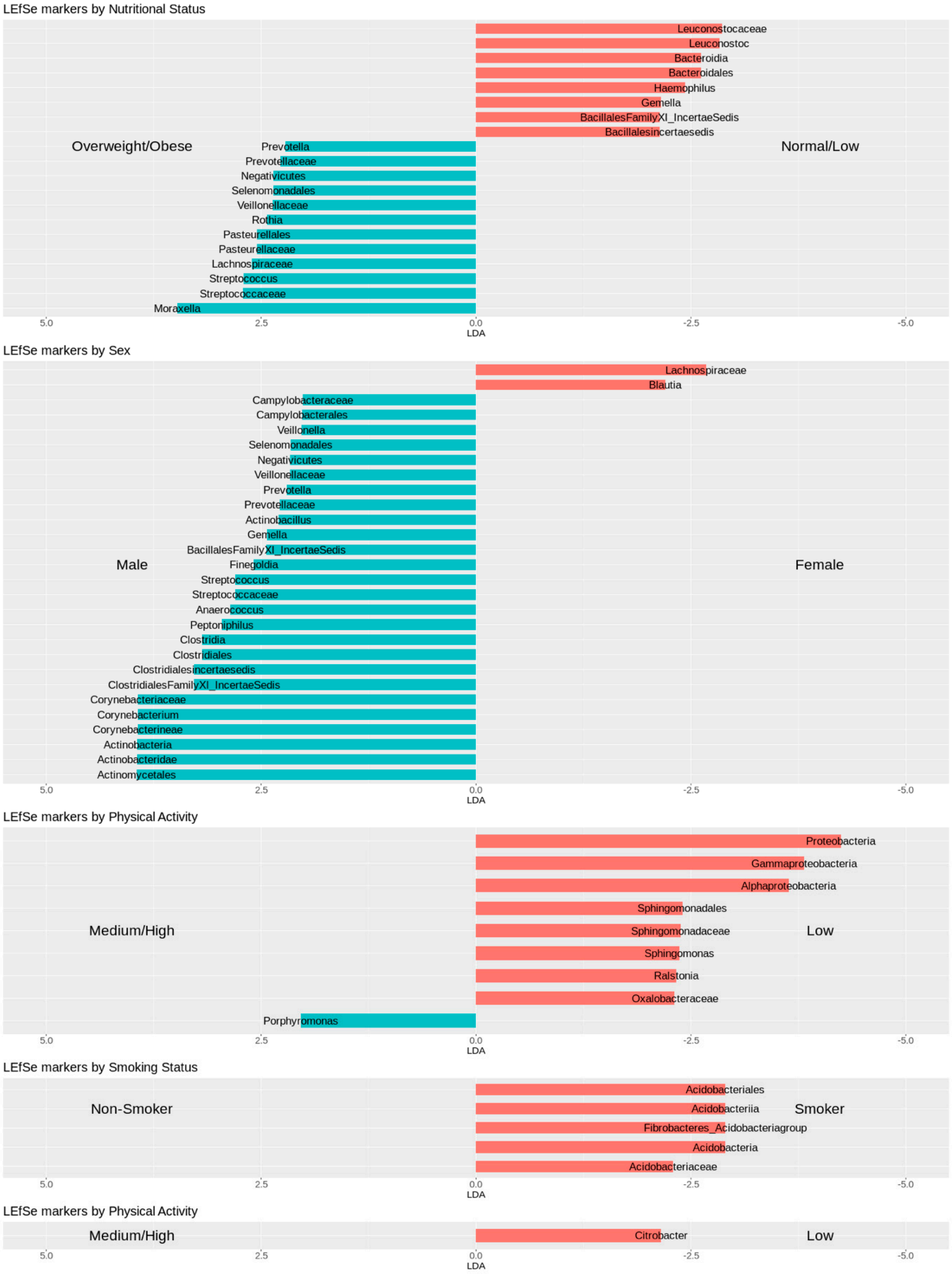


**Figure S3.** Marker sets found for general comparisons among different dichotomies from metadata categories from the adult samples.

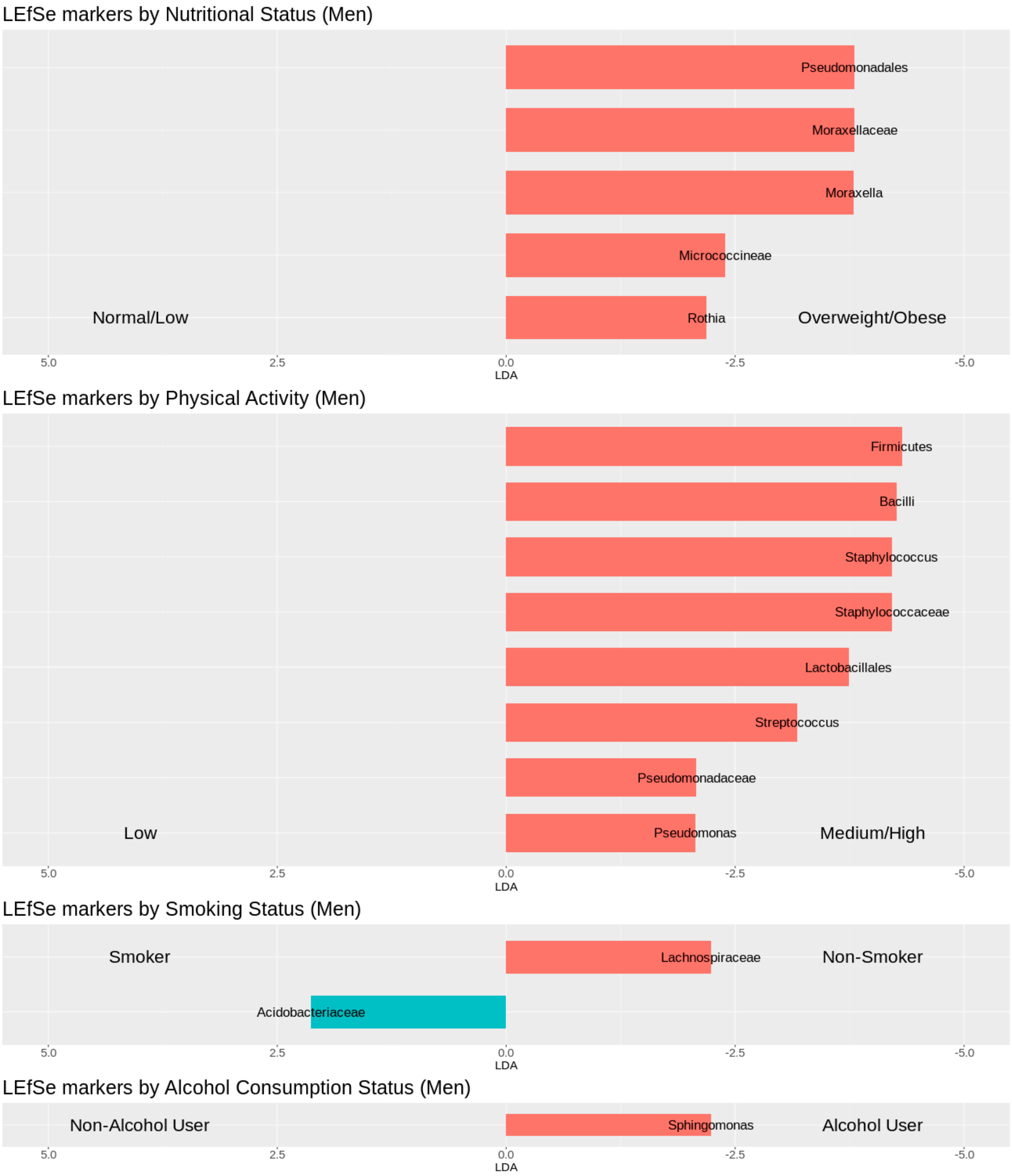


**Figure S4.** Marker sets are found for comparisons among different dichotomies from metadata categories from the adult male samples.

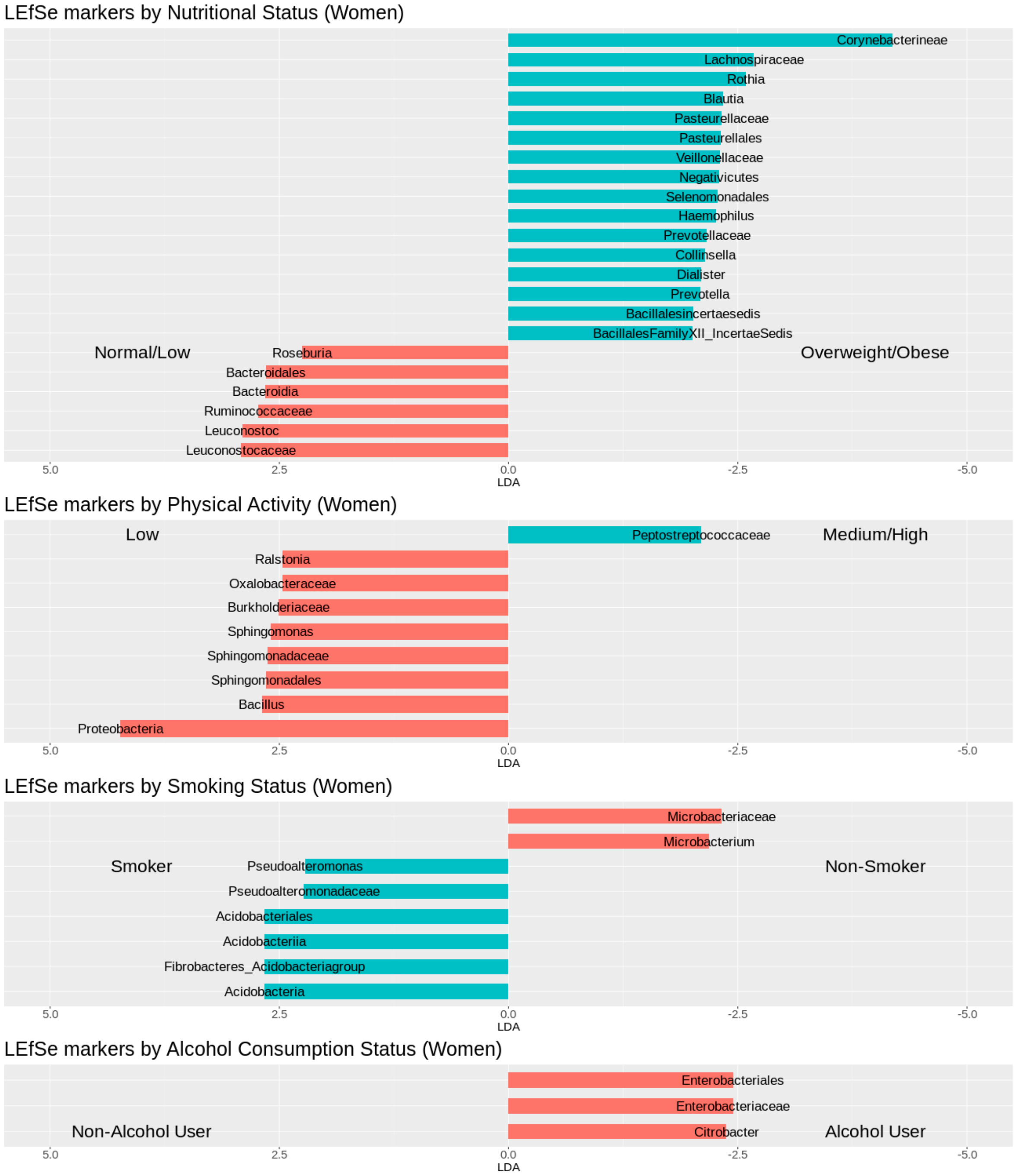


**Figure S5.** Marker sets are found for comparisons among different dichotomies from metadata categories from the adult female samples.

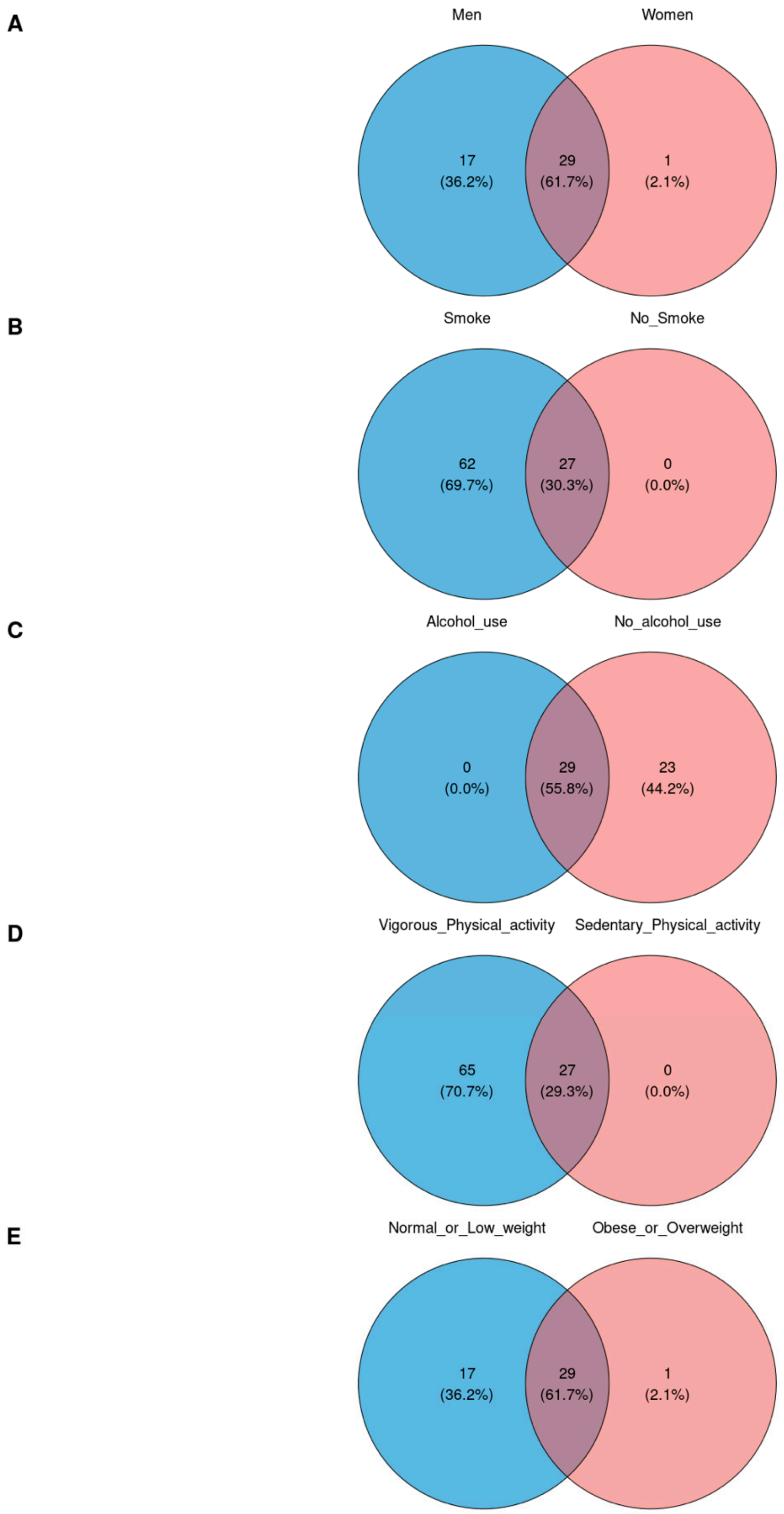


**Figure S6.** Venn Diagrams representing the differences across "top prevalent groups" between samples across different host properties and lifestyles. (A) sex, (B) smoking, (C) alcohol use, (D) physical activity and (E) nutritional status.

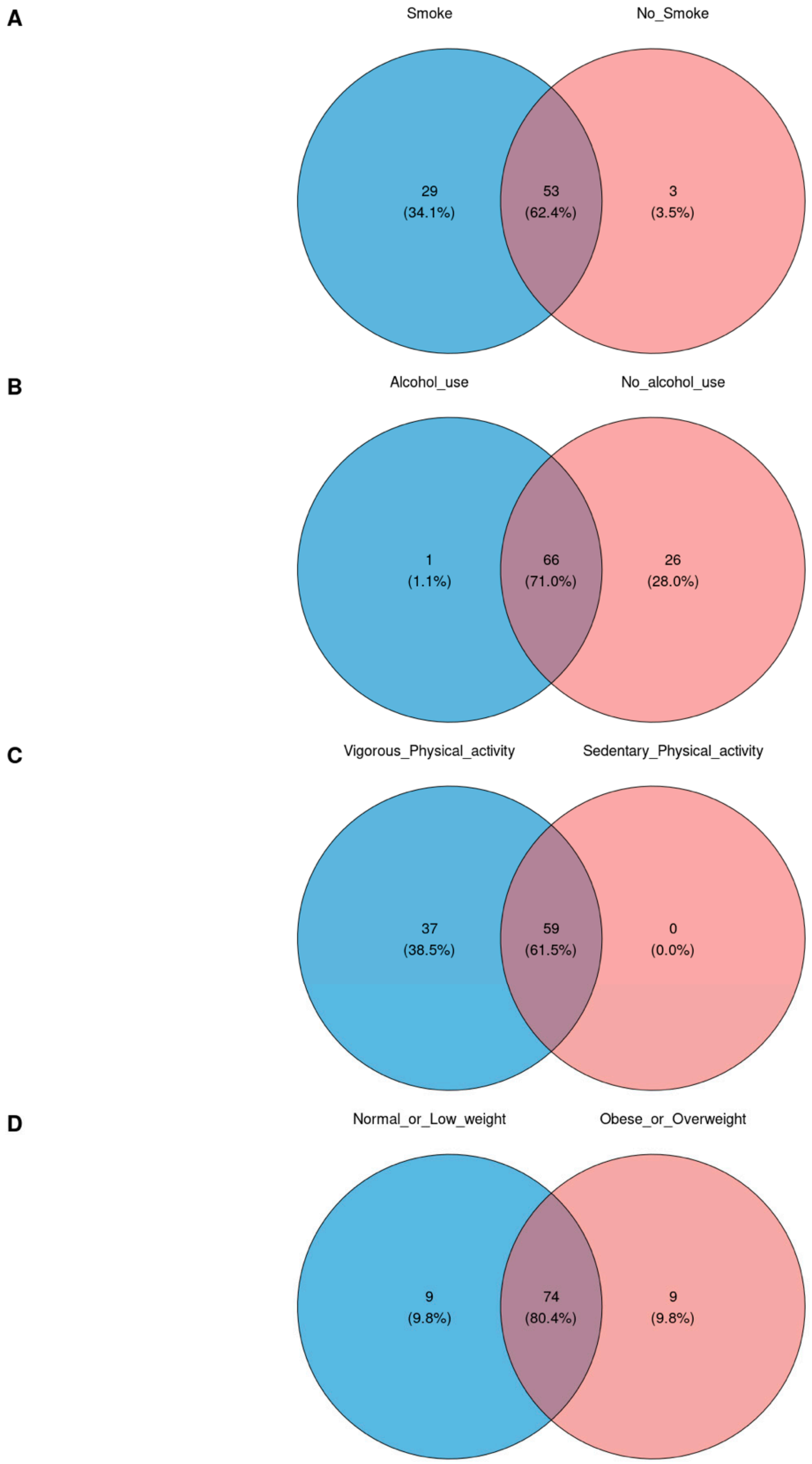


**Figure S7.** Venn Diagrams representing the differences across "top prevalent groups" between samples across different host lifestyles in men samples. (A) smoking, (B) alcohol use, (C) physical activity and (D) nutritional status.

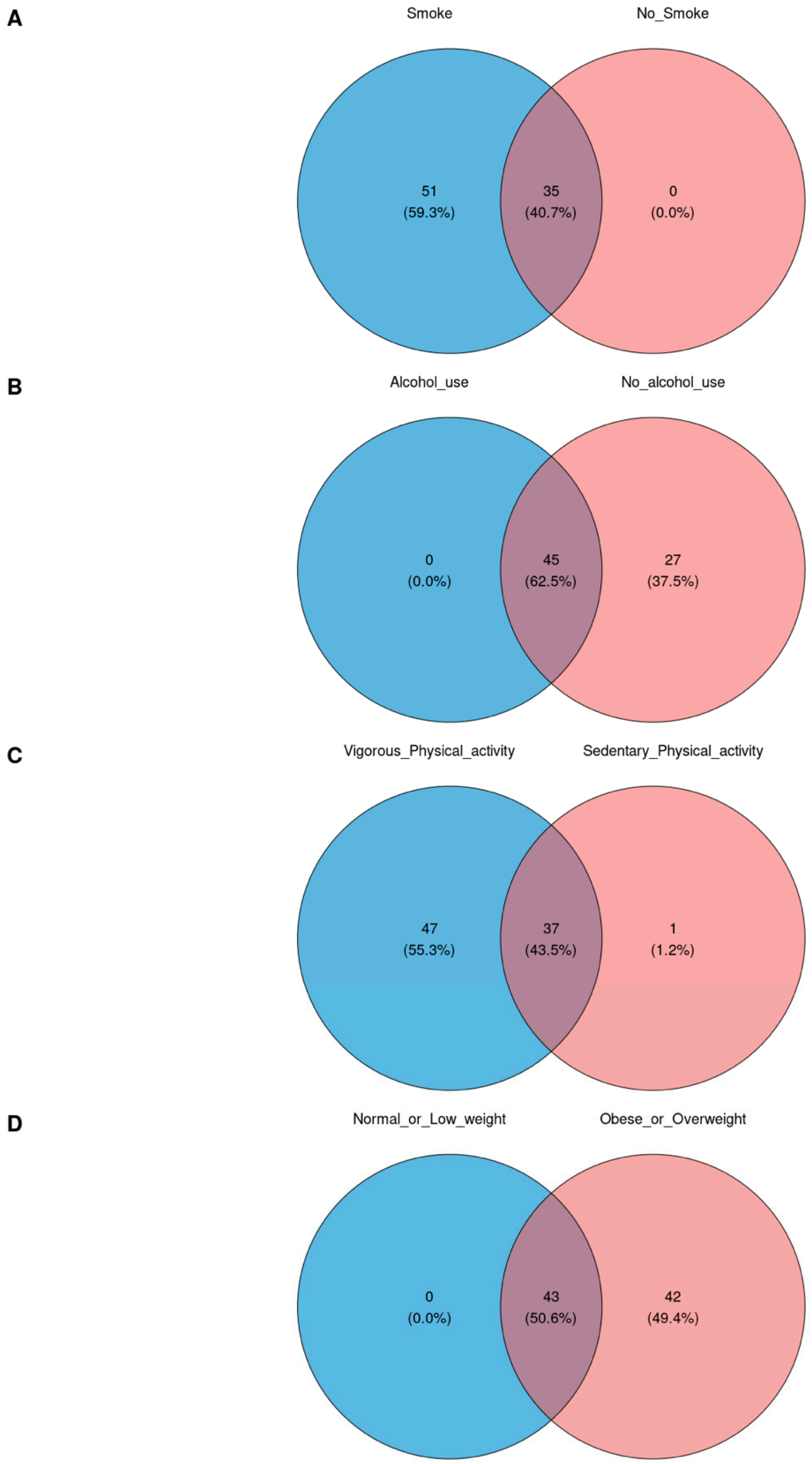


**Figure S8.** Venn Diagrams representing the differences across "top prevalent groups" between samples across different host lifestyles in women samples. (A) smoking, (B) alcohol use, (C) physical activity and (D) nutritional status.

*Supplementary Tables*

**Table S1.** Descriptive statistics of alpha diversity indexes of microbiota and lifestyle attributes of the participants (n=110).

| Variable | Definition | Value |
|---|---|---|
| Shannon's Index | Entropy Shannon's index, also called Shannon-Wiener Index, measures the evenness of taxonomic groups in the sample, in mean (sdt. dev.) | 2.84 (0.3) |
| Equivalent number index | Equivalent number index, or true diversity, measures the effective number of taxonomic groups in the sample, mean (sdt. dev.) | 17.7 (4.7) |
| Simpson's index | Probability that two units randomly selected from a sample will belong to the same taxonomic group, in mean (sdt. dev.) | 0.1 (0.02) |
| Inverse of Simpson | Inverse of Simpson, or 1/Simpson's index, measures the richness of taxonomic groups in the sample, in mean (sdt. dev.) | 12.9 (2.8) |
| Physical Activity | Self-reported physical activity of individuals, classified in three levels: sedentary, active, and vigorous, in n (%) | |
| | i) Sedentary, or light activity lifestyles, include people with occupations that do not demand much physical effort, are not required to walk long distances, use motor vehicles for transportation, and spend most of their leisure time sitting or standing, with little body displacement. Examples: office workers, people selling produce at home or in the marketplace or doing light household chores and caring for children in or around their houses. | 70 (64) |
| | ii) Active, or moderately active lifestyles, refers to people with occupations that are not strenuous in terms of energy demands, but involve more energy expenditure than that described for sedentary lifestyles. This includes individuals with sedentary occupation who regularly spend a certain amount of time in moderate to vigorous physical activities. Examples: the daily performance of one hour (either continuous or in several bouts during the day) of moderate to vigorous exercise, such as jogging/running, cycling, aerobic dancing or various sports activities; also, occupations such as masons and construction workers. | 19 (17) |
| | iii) Vigorous, or vigorously active lifestyles, includes people engaging regularly in strenuous work or in strenuous leisure activities for several hours, for example: people with occupations such as swimming or dancing an average of two hours each day, or non-mechanized agricultural laborers. | 21 (19) |
| Nutritional status | Nutritional status was determined by self-reported height and weight, to calculate the individual's BMI (body mass index) and stratified in categories normal weight, overweight, and obesity, in n (%). | |
| | i) Normal weight (BMI: 18.5–24.9 kg/m2) | 66 (60) |
| | ii) Overweight (BMI: 20.5–29.9 kg/m2) | 36 (33) |
| | iii) Obesity (BMI: ≥30.0 kg/m2) | 8 (7) |
| Smoking | Individual report of being smoker (yes=1), in n (%) | 18 (16) |
| Interaction between nutritional status and smoking | Normal weight and smoking, n(%) | 12 (11) |
| | Overweight and smoking, n (%) | 4 (4) |
| | Obese and smoking, n (%) | 2 (2) |
| Medication | Individual report of frequent medication, in n(%) | 38 (35) |
| Consumption of Alcohol | Self-reported consumption of alcohol, in n(%) | 77(70) |
| Female | Sex of individuals (female=1), in n(%) | 63 (57) |
| Age | Self-reported age of individuals, in years. Mean (std. dev.) | 33.7 (10.9) |

BMI: body mass index.

**Table S2.** Associations between alpha diversity of microbiota and lifestyle attributes of individuals with normal weight (n=66) and overweight-obese (n=44).

| Variables | Alpha diversity indexes | | | | | | | |
|---|---|---|---|---|---|---|---|---|
| | Shannon's | | Equivalent number | | Simpson | | Inverse of Simpson | |
| | Normal weight | Overweight-obese | Normal weight | Overweight-obese | Normal weight | Overweight-obese | Normal weight | Overweight-obese |
| Physical activity | | | | | | | | |
| Sedentary vs active | −0.29** | 0.03 | −5.18** | 0.22 | 0.02** | −0.00 | −3.28** | 0.16 |
| | (−0.44, −0.14) | (−0.27, 0.33) | (−7.67, −2.68) | (−5.29, 5.72) | (0.01, 0.03) | (−0.02, 0.02) | (−4.79, −1.77) | (−2.78, 3.11) |
| Vigorous vs active | −0.23* | −0.09 | −4.16* | −1.60 | 0.01 | 0.01 | −2.25* | −1.08 |
| | (−0.45, −0.01) | (−0.47, 0.28) | (−7.76, −0.56) | (−8.34, 5.15) | (−0.00, 0.03) | (−0.02, 0.04) | (−4.41, −0.09) | (−4.78, 2.61) |
| Nutritional status | ^ | ^ | ^ | ^ | ^ | ^ | ^ | ^ |
| Smoking | −0.18** | 0.10 | −3.21** | 2.00 | 0.01* | −0.01 | −1.59* | 1.00 |
| | (−0.32, −0.05) | (−0.17, 0.38) | (−5.48, −0.95) | (−3.75, 7.75) | (−0.00, 0.02) | (−0.02, 0.01) | (−2.98, −0.20) | (−1.59, 3.59) |
| Interaction between nutritional status and smoking | ^ | ^ | ^ | ^ | ^ | ^ | ^ | ^ |
| Consumption of alcohol | −0.16* | 0.20* | −2.51* | 3.34* | 0.02** | −0.01 | −2.04** | 1.73 |
| | (−0.31, −0.02) | (0.00, 0.39) | (−4.88, −0.13) | (0.25, 6.43) | (0.01, 0.03) | (−0.03, 0.00) | (−3.44, −0.65) | (−0.08, 3.55) |

Note: Cells show ordinary least square coefficients and, in parenthesis, the confidence interval at 95%. Coefficients report the change in the index of alpha diversity for a unit change in the lifestyle attribute. Statistically insignificant variables were omitted from the table, such as female, medication, age, and square of age. ^ refers to variables omitted in the analysis. ** and * refer to p<0.01 and p<0.05, respectively.

**Table S3.** Associations between alpha diversity of microbiota and lifestyle attributes among healthy adults (n=110).

| Variables | Alpha diversity indexes | | | |
|---|---|---|---|---|
| | Shannon's | Equivalent number | Simpson | Inverse of Simpson |
| Physical activity | | | | |
| Sedentary vs active | −0.16* | −2.91* | 0.01 | −1.78* |
| | (−0.32, 0.00) | (−5.65, −0.16) | (<0.00, 0.02) | (−3.36, −0.20) |
| Vigorous vs active | −0.19* | −3.38* | 0.01 | −1.94* |

| | | | | |
|---|---|---|---|---|
| | (−0.39 – >0.00) | (−6.66, −0.09) | (<0.00 – 0.03) | (−3.86, −0.02) |
| Nutritional status | | | | |
| Overweight vs normal weight | −0.02 | −0.44 | >0.00 | −0.24 |
| | (−0.16, 0.12) | (−2.69, 1.81) | (−0.01, 0.01) | (−1.53, 1.05) |
| Obese vs normal weight | 0.01 | 0.08 | <0.00 | −0.08 |
| | (−0.23, 0.25) | (−4.04, 4.21) | (−0.02, 0.01) | (−2.41, 2.25) |
| Smoking | −0.17* | −3.02* | 0.01 | −1.40 |
| | (−0.32, −0.02) | (−5.53, −0.52) | (<0.00, 0.02) | (−2.96, 0.16) |
| Interaction between nutritional status and smoking | | | | |
| Overweight and smoking | 0.34* | 6.11 | −0.02* | 3.20* |
| | (0.03, 0.66) | (−0.30, 12.52) | (−0.04, <0.00) | (0.01, 6.39) |
| Obese and smoking | 0.22 | 3.95 | −0.01 | 1.17 |
| | (−0.27, 0.71) | (−4.93, 12.83) | (−0.04, 0.02) | (−3.26, 5.60) |

Note: Cells show ordinary least square coefficients and, in parenthesis, the confidence interval at 95%. No statistically significant variables were omitted from the table, such as female, consumption of alcohol, medication, age, and square of age. ^ refers to variables omitted in the analysis. ** and * refer to p<0.01 and p<0.05, respectively.
